# What Are the Boundaries for Defining Gender?

**DOI:** 10.1007/s10508-025-03309-w

**Published:** 2025-09-20

**Authors:** Bruce M. King

**Affiliations:** Department of Psychology, Clemson University, Clemson, SC 29634 USA

In 2010, I authored an editorial published in *American Journal of Physiology* that attempted to educate physiology and other biomedical researchers about the proper usage of the terms “sex” and “gender” (King, 2010). Using the Medline database, I had found that in the period 2005 to 2009 there had been 197 published papers that had used the key words “gender differences” and “rats” together, with 107 of them using “gender” in the title. The number no doubt would have been greater had the search included other nonhuman research species (e.g., mice). Using Science Citation Index, Haig (2004) had previously found that in the titles of academic papers published in the natural sciences in 2000–2001 (not social sciences, arts, and humanities) there was “more than 1 use of gender for every 2 uses of sex.”

Many of the published physiology papers that I examined had nothing to do with behavior. For example, article titles included “gender differences” for “GABAA receptor-mediated postsynaptic currents” (Chudomel et al., 2009), “erythrocyte and brain decosahexaenoic acid composition” (McNamara et al., 2009), “liver and kidney expression of sulfate anion transporter sat-1” (Brzica et al., 2009), “proliferation and osteogenic differentiation of bone marrow stromal cells” (Hong et al., 2009), and “beta-adrenergic receptor responsiveness” (Bilginoglu et al., 2007).

During this same period (2005 to 2009), sports journalists reported gender differences between fillies and mares (horse racing), and there had been many articles about a human track star who had been required to take a urine test to prove her gender. While forensic anthropologists continue to debate whether biological sex can unequivocally be determined by examining bones (Weiss, 2024), another newspaper article reported about an autopsy being performed on human skeletal remains to determine the victim’s gender.

In this set of commentaries, there will be debate about how many genders there are among humans. I admit that I do not know the answer. However, I hope we all can agree that nonhuman species do not possess gender and that, among humans, one’s gender cannot be determined by an autopsy or anatomical exam.

The origin of the use of the term gender in academic research has been attributed to a 1955 paper by John Money in which he investigated how people with ambiguous genitalia self-identified (Haig, 2023; see Haig, 2004, for an excellent review). The term was subsequently used widely by feminist scholars in the 1970s to differentiate the socialization experiences of men and women from the biological differences or the act of having sex (e.g., Gould & Kerr-Daniels, 1977; Kessler & McKenna, 1978; Unger, 1979). The American Psychological Association (2001) officially endorsed this distinction in 1991: “Gender is cultural and is the term to use when referring to men and women as social groups. Sex is biological; use it when biological distinction is prominent” (p. 63). By this definition, it is inappropriate to use “gender” to categorize human subjects in medical studies of such things as cancer, heart or liver disease, or response to new medications. As stated by Golden (2000), gender is not something that resides inside a person, but is used “only when discussing social, cultural, and psychological aspects that pertain to the traits, norm, stereotypes and roles considered typical and desirable for those whom society has designated as male or female” (p. 33).

One of the major reasons that researchers using nonhuman animals as subjects have given for using the term “gender” is “a desire to signal sympathy with feminist goals” (Haig, 2004, pp. 94–95). However, the usage of “sex” and “gender” interchangeably is precisely the opposite of what feminist scholars intended and as Zucker (2025) stated in his invitation to submit commentaries, this suggests that politics has increasingly played a role in the use of “gender.”

The role of sexual politics is apparent even when referring to one’s biological sex. For example, in the 1900s researchers and practitioners used the terms “hermaphrodite” and “pseudohermaphrodite” to refer to individuals with both male and female reproductive anatomy or who were born with ambiguous genitalia. These were the widely accepted medical terms of that era, with no intended negative connotations (e.g., see Money, 1955). The terms were derived from the Greek mythological figure Hermaphroditus who merged with the nymph Salmacis, resulting in a body with both male and female genitalia. However, by the mid-1990s some people began to feel that these terms were disparaging and stigmatizing, and replaced them with the terms “intersexual individuals” or “intersexuals” to refer to a wide variety of chromosome or hormonal abnormalities (see Chase, 1998).

By 2010, others felt that the term “intersexual” was also disparaging and stigmatizing. Papers were published stating that the preferred term by both health professionals and parents was “disorders of sex development” (Davies et al., 2011; Dreger et al., 2005; Hughes et al., 2006; Marino, 2010). By 2018, still others were objecting to the word “disorders.” A Consensus Panel of European multidisciplinary experts and patient representatives advocated for “differences of sex development” (DSD) or even a return to “intersexual” (Cools et al., 2018). Other patient support groups also preferred “intersex” (e.g., Babu, 2022). However well-meaning these attempts were to find terms acceptable to everyone, disagreements in use of nomenclature in academics can only lead to confusion among the general public.

In the present debate, I urge that our usage of the term “gender” (vs. sex) be in accord with APA guidelines: “unambiguous” (APA, 2001). No matter how many genders any scholar proposes, none should be used just to please certain constituencies or as a synonym for “sex,” but should “distinguish culturally specific characteristics of masculinity and femininity from biological factors” (Hawkesworth, 1997).
